# U-Shaped Association Between Serum Uric Acid Level and Hypertensive Heart Failure: A Genetic Matching Case-Control Study

**DOI:** 10.3389/fcvm.2021.708581

**Published:** 2021-12-08

**Authors:** Hongxuan Xu, Quan Wang, Yunqing Liu, Lingbing Meng, Huanyu Long, Li Wang, Deping Liu

**Affiliations:** ^1^Chinese Academy of Medical Sciences, National Center of Gerontology, National Health Commission, Department of Cardiology, Beijing Hospital, Institute of Geriatric Medicine, Beijing, China; ^2^The Key Laboratory of Geriatrics, Chinese Academy of Medical Sciences, Beijing Institute of Geriatrics, Beijing Hospital National Center of Gerontology, Institute of Geriatric Medicine, Beijing, China; ^3^Yuetan Community Health Center, Fuxing Hospital, Capital Medical University, Beijing, China; ^4^Chinese Academy of Medical Science, Peking Union Medical College, Beijing, China; ^5^Chinese Academy of Medical Sciences, Departments of Neurology, National Center of Gerontology, Beijing Hospital, Institute of Geriatric Medicine, Beijing, China; ^6^Peking University Health Science Centre, Peking University Fifth School of Clinical Medicine, Beijing, China

**Keywords:** uric acid, heart failure, matched, lipids, case-control

## Abstract

**Background:** Heart failure (HF) is a global pandemic and lays an added burden on public healthcare. Previous studies indicated that high and low serum uric acid levels are associated with worse outcomes in many diseases. Reduced serum uric acid may not result in a better outcome.

**Methods:** A comparative, matched cross-sectional study design was implemented. The matching variables were age, sex, BMI, BP, and histories of CKD, CVD, diabetes mellitus, stroke, hyperlipidemia. We reviewed the electronic medical records to identify patients diagnosed with hypertension or hypertensive heart failure (HHF) admitted to Beijing Hospital's cardiology department.

**Results:** The median age of the two groups after matching are 71. There are 55.6% males in the hypertension group and 53.8% in the heart failure group. Univariate logistic regression analysis showed that UA's quadratic term is significant (*OR* = 1.00, 95% CI 1.00 to 1.00; *P* = 0.03), which indicated a u-shaped relationship between hypertension and HHF. FBS (*OR* = 0.22, 95% CI 0.05 to 0.95, *p* = 0.07) and HDL (*OR* = 1.23, 95% CI 1.00 to 1.54, *P* = 0.05) were not significant but close.

**Conclusion:** Our results supported that both low and high uric acid levels were predictive of hypertensive heart failure. Besides, high-density lipoprotein cholesterol and fasting blood sugar were also associated with hypertensive heart failure. Low-density lipoprotein cholesterol was not associated with hypertensive heart failure.

## Introduction

Heart Failure (HF) is a global pandemic and lays an added burden on public healthcare. It affects ~13.5 million people aged over 35 years in China and results in 1 million hospitalizations annually in the United States and Europe (1, 2). HF is a shared end-stage phase of many cardiac diseases; therefore, HF patients can have mixed etiologies. Hypertension is probably one of the most common risk factors for developing HF. As proof, in the Framingham Heart Study, 91% of participants' hypertension antedated HF development. Multivariable analyses indicated that hypertension accounts for 39% of HF cases in men and 59% in women (3).

Persistent hypertension can lead to HF with normal ejection fraction or HF with reduced ejection fraction, the latter mainly through myocardial infarction (4). Chronically increased stress and neurohormones imposed on the cardiac myocytes caused by high blood pressure (BP) will result in left ventricular hypertrophy, diastolic dysfunction, and eventually hypertensive heart failure (HHF). Thus, HHF is characterized by myocardial abnormality resulting from long-standing arterial hypertension in the absence of any other cardiac disease that can cause LVH or cardiac dysfunction. Progressive extracellular matrix (ECM) accumulation is part of the underlying pathophysiological mechanism for heart failure in patients with pressure overload hypertrophy (POH) (5, 6).

Researchers have focused on detecting predictors and hypothesizing the underlying HF pathophysiology. Elevated serum uric acid (SUA) is a marker of inflammatory cytokine activation, insulin resistance, and oxidative stress (7). In addition, hyperuricemia may directly contribute to worsening HF outcomes (8, 9). Dyslipidemia, hyperglycemia, and hyperuricemia are widely discussed risk factors or markers of cardiovascular diseases (CVD). The so-called reverse epidemiology of HF has been introduced for years, that is risk factors in the general population, including body mass index (BMI), serum cholesterol, and BP, are positively related to a better outcome in patients with chronic heart failure (CHF) (10).

From this perspective, this study aims to explore the causal relation of uric acid with hypertensive patients developing HHF while adjusting covariates like lipoproteins and blood sugar.

## Methods

### Design and Population

This single-center, matched, comparative case-control study was conducted at Beijing Hospital. We reviewed the electronic medical records to identify patients diagnosed with hypertension or HHF admitted to Beijing Hospital's cardiology department during Jan 1, 2017, and Dec 31, 2017. Hypertension is defined as BP ≥ 140 mmHg systolic or 90 mmHg diastolic (11). Due to the universally accepted definition of HHF is unavailable, we set our inclusion criteria for the selection of HHF patients as (a) hypertensive patients; (b) left ventricular alteration by an echocardiographic assessment or Cardiac magnetic resonance imaging; (c) New York Heart Association (NYHA) Classification ≥ Class II. The exclusion criteria were patients with (a) chronic respiratory diseases; (b) hyperthyroidism or hypothyroidism; (c) cancer; (d) autoimmune disorders; (e) valvular heart diseases. The actual sample sizes of pre-matching participants were 265 and 264, respectively.

### Data Collection

We collected demographic characteristics, biochemical tests, and BP from the electronic medical record system. Blood samples were obtained after at least 8-h fasting the following morning after admission. Demographic characteristics included age; gender; body mass index (BMI); history of diabetes mellitus, hyperlipidemia, cardiovascular diseases (CVD), chronic kidney diseases (CKD), and stroke. Biochemical tests included fasting blood sugar (FBS), triglycerides (TG); total cholesterol (TC); high-density lipoprotein cholesterol (HDL-c); low-density lipoprotein cholesterol (LDL-C); serum uric acid (SUA), and creatinine (Cr).

### Genetic Matching

To control potential confounding covariates and underlying bias, researchers have utilized matching in many fields as a method of causal inference. Propensity score and Mahalanobis distance (MD) matching are currently the most popular methods for matching. However, if the covariates have non-ellipsoidal distributions, MD and a misspecified propensity score model do not perform well (12, 13). Genetic matching is a generalization of propensity score and MD, developed by Sekhon and Mebane using an evolutionary search algorithm to maximize the balance of observed covariates across matched treated and control units (14). Genetic Matching matches by minimizing a generalized version of Mahalanobis distance (GMD) that is:


(1) $$GMD\left(X_{i},X_{j},W\right)=\sqrt{{\left(X_{i}-X_{j}\right)}^{T}{\left(S^{-\frac{1}{2}}\right)}^{T}WS^{-\frac{1}{2}}(X_{i}-\;X_{j})},$$

W is a k × k positive definite weight matrix, and $S^{-\frac{1}{2}}$ is the Cholesky decomposition of S.

The matching variables were age, sex, BMI, BP, and histories of CKD, CVD, diabetes mellitus, stroke, hyperlipidemia.

### Statistical Analysis

All data were analyzed using R 4.0.5. The major packages used for matching were “matching,” “cobalt,” and “matchit.” Comparisons between 2 groups of continuous variables were assessed for statistical significance by unpaired Student *t*-test or *z*-test, and binary variables by Pearson's χ^2^ test. Potential risk factors were first analyzed by univariable logistic regression analysis. Statistically significant predictors were further analyzed for their independent predictive value using multivariable logistic modeling. We used the Hosmer-Lemeshow tests to test the goodness of fit of logistic regression models. Likelihood ratio (LR χ^2^) tests were used to compare the fitness of two statistical models. The significance level was defined as *P* < 0.05. All the hypothesis tests were 2-sided.

## Results

### Characteristics and Genetic Matching

**Figure 1:**
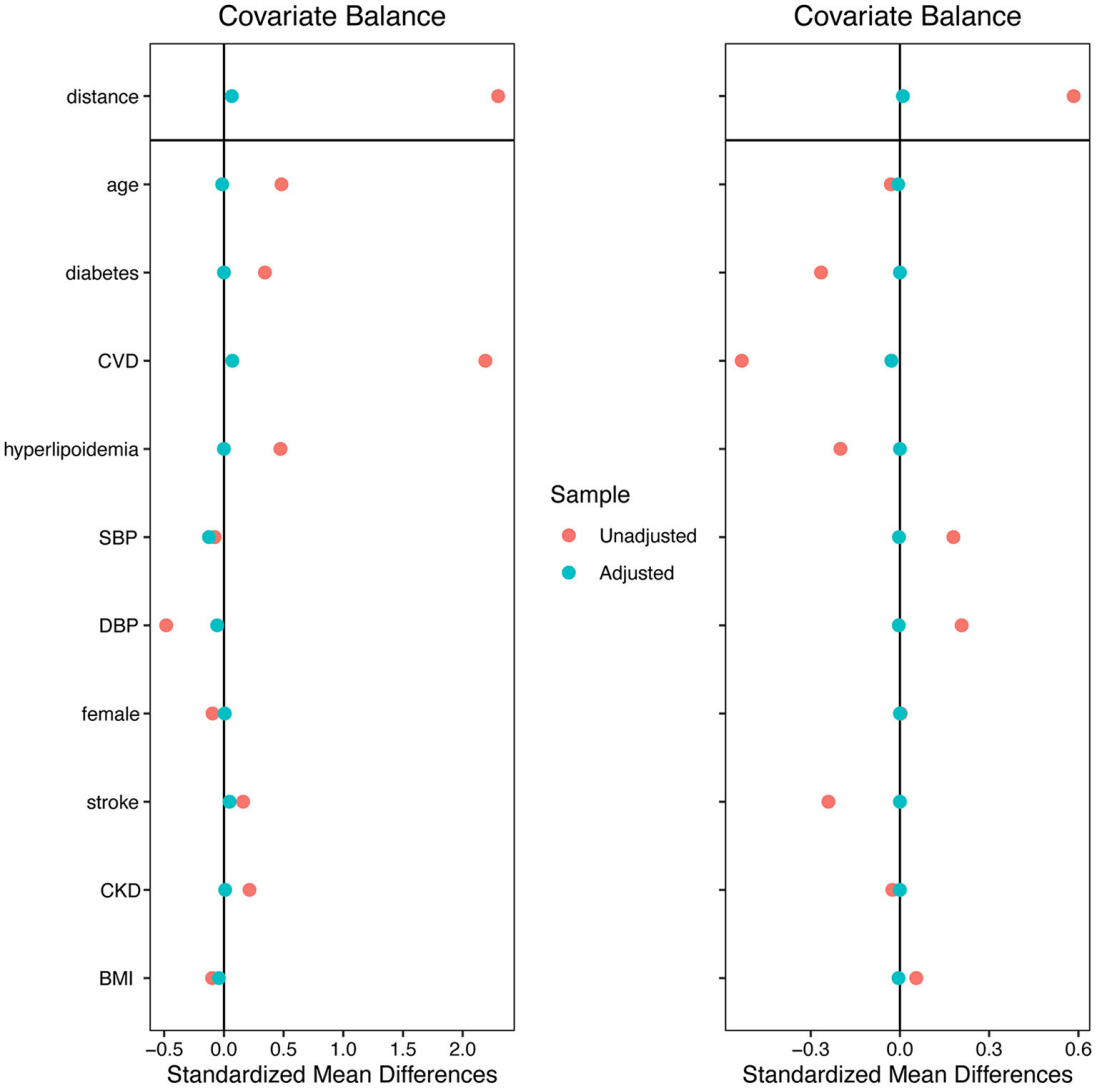
Love-plots with the balance statistic on the X-axis of each matching.

A total of 529 individuals were selected from all 2,722 records, 265 were included in the hypertension group and 264 in the HHF group to perform genetic matching. After 1:1 matching twice, 137 individuals were successfully matched. Table 1 showed the essential demographic characteristics and medical histories before and after matching. The difference of all covariates between the two groups after matching reached insignificance, indicating that genetic matching yields an equilibrious distribution of covariates, leading to a comparable paired dataset. Figure 1 presented two Love-plots (left was the first matching and right the second) with the balance statistic on the X-axis of each matching, all the points after adjustment were within the 0.1 standardized mean difference threshold. It also was good evidence that balance had been achieved. We exchanged the data between both groups to perform the second matching and exchanged it back after matching, so the red points of the right plot are vertically flipped regarding the green points of the left. Table 2 summarized the potential risk factors examined in this study.

**Table 1:** Demographic characteristics.

	**Before matching**			**After matching**		
**Clinical Values**	**Hypertension (*N* = 265)**	**HHF (*N* = 264)**	***P*-value**	**Hypertension (*N* = 72)**	**HHF (*N* = 65)**	***P*-value**
Age, median (IQR)	66 [26, 96]	72 [37, 91]	<0.001	71 [53, 91]	71 [37, 88]	0.88
Male No. (%)	135 (51%)	147 (56%)	0.315	40 (56%)	35 (54%)	0.977
BMI	26 (16, 38)	25 (17, 37)	0.284	25.7 (4)	25.8 (4)	0.897
Blood pressure, mean (SD), mmHg
Systolic	143 (21)	141 (20)	0.38	145 (18)	144 (21)	0.968
Diastolic	82 (14)	76 (12)	<0.001	78 (11)	78 (11)	0.964
Diabetes No. (%)	80 (30%)	125 (47%)	<0.001	25 (35%)	25 (39%)	0.782
CVD No. (%)	47 (18%)	233 (88%)	<0.001	45 (63%)	44 (68%)	0.648
Hyperlipidemia No. (%)	231 (87%)	254 (96%)	<0.001	65 (90%)	60 (92%)	0.907
Stroke No. (%)	37 (14%)	54 (21%)	0.062	9 (13%)	9 (14%)	>0.99
CKD No. (%)	19 (7%)	39 (15%)	0.008	10 (14%)	10 (15%)	>0.99

*BMI, body mass index; CVD, cardiovascular diseases; CKD, chronic kidney diseases*.

**Table 2:** The potential risk factors.

	**Before matching**		**After matching**	
	**Hypertension (*N* = 265)**	**HHF (*N* = 264)**	**Hypertension (*N* = 72)**	**HHF (*N* = 65)**
FBS mmol/L	5.40 [2.80, 21.1]	5.80 [2.37, 24.9]	5.55 [2.80, 8.90]	5.70 [4.00, 15.1]
Cr μmol/L	68 [30, 42]	75 [34, 52]	70 [39, 22]	70 [43, 26]
SUA μmol/L	337 ± 88.5	351 ± 106	341 ± 86.7	363 ± 124
TC mmol/L	4.21 [2.09, 8.89]	3.58 [1.44, 7.78]	3.93 [2.34, 8.89]	3.79 [2.39, 6.81]
TG mmol/L	1.31 [0.47, 14.40]	1.32 [0.40, 14.40]	1.30 [0.52, 4.49]	1.35 [0.40, 8.07]
HDLC mmol/L	1.19 ± 0.83	1.09 ± 0.66	1.14 ± 0.28	1.06 ± 0.20
LDLC mmol/L	2.65 ± 1.89	2.21 ± 0.84	2.47 ± 0.89	2.38 ± 0.82

*FBS, fasting blood sugar; TG, triglycerides; TC, total cholesterol; HDLC, high-density lipoprotein cholesterol; LDLC, low-density lipoprotein cholesterol; SUA, serum uric acid; Cr, creatinine*.

### Logistic Regression

**Figure 2:**
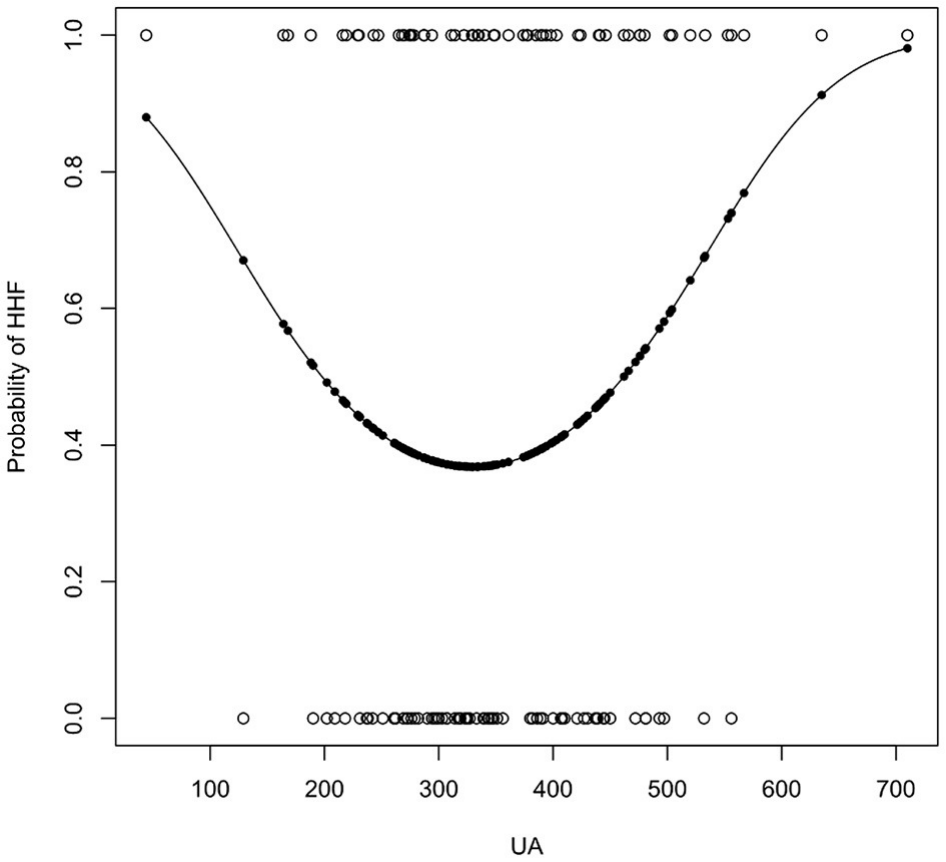
The result of univariate logistic regression of UA. The x-axis is the concentration of uric acid (μmol/L).

Univariate logistic regression analysis (Table 3) showed that UA's quadratic term is significant (*OR* = 1.00, 95% CI −1.00 to 1.00; *P* = 0.03), which indicated a u-shaped relationship between hypertension and HHF. FBS (OR = 0.22, 95% CI 0.05 to 0.95, *p* = 0.07) and HDL (*OR* = 1.23, 95% CI 1.00 to 1.54, *P* = 0.05) were statistically not significant but close. The confidence intervals of the odds ratio (OR) of both were either above or below 1. For 1 unit increased in FBS, the odds of developing HHF increased by a factor of 1.23. For 1 unit increased in HDL-c, the odds of developing HHF were 4.55 times lesser. Figure 2 presented the relationships between UA and the probability of developing HHF. The probability of HHF regarding SUA was lowest at around 200–300μmol/L, lower or higher uric acid concentrations were both positively associated with the incidence of HHF.

**Table 3:** Logistic regression results.

	**B**	**SE**	**WALD**	***P*-value**	**OR (95%CI)**
**Univariate Logistic Regression**					
TC	−0.1444	0.1735	0.692	0.405	0.87 [0.61, 1.21]
TG	0.2557	0.2062	1.540	0.210	1.29 [0.88, 2.01]
LDL-c	−0.1279	0.2063	0.384	0.535	0.88 [0.58, 1.31]
HDL-c	−1.4963	0.7727	3.748	0.053	0.22 [0.05, 0.95]
FBS	0.2030	0.1102	3.397	0.065	1.23 [1.00, 1,54]
Cr	0.006810	0.005055	1.814	0.178	1.01 [1.00, 1.02]
UA	−2.044E-02	1.055E-02	3.752	0.053	0.98 [0.96, 1.00]
UA * UA	3.098E-05	1.453E-05	4.545	0.033	1.00 [1.00, 1.00]
**Multivariate Logistic Regression**					
UA	−2.204E-02	1.127E-02	3.830	0.0504	0.98 [0.95, 1.00]
UA * UA	3.232E-05	1.525E-05	4.490	0.0341	1.00 [1.00, 1.00]
HDL-c	1.983E-01	1.129E-01	3.087	0.0790	0.24 [0.04, 1.09]
FBS	−1.437E+00	8.091E-01	3.154	0.0757	1.22 [0.99, 1.55]

*FBS, fasting blood sugar; TG, triglycerides; TC, total cholesterol; HDLC, high-density lipoprotein cholesterol; LDLC, low-density lipoprotein cholesterol; SUA, serum uric acid; Cr, creatinine*.

The multivariate logistic regression analysis results, including FBS, HDL, and UA, were similar to the univariate logistic regression analysis Table 3. The Hosmer-Lemeshow (χ^2^ = 6.48, *df* = 8, *p* = 0.59) tests indicated evidence of a good fit model. Moreover, the likelihood ratio test compared the multivariate logistic regression model with the model without predictors yielded a significant result (LR χ^2^ = 14.93, *df* = 4, *p* = 0.005).

We then tested the interactions among variables. The interaction between FBS and UA in the multivariate logistic regression analysis was not significant (*OR* = 0.99, *p* = 0.08). The likelihood ratio test compared the multivariate model with the model without interaction yielded a not significant result (*p* = 0.07).

## Discussion

In the present study, we examined the association with multiple variates to patients with HHF. The results demonstrated that low and high serum UA levels were significantly associated with the probability of developing HHF, *videlicet* the U-shaped association between serum UA levels and HHF. On the other hand, HDL was negatively associated with HHF.

### Serum Uric Acid

Uric acid is correlated with blood pressure in children with new-onset, untreated, primary hypertension, 90% of newly diagnosed adolescent hypertensives are hyperuricemic (15). Urate lowering therapies may mitigate hypertension caused by hyperuricemia (16).

It is still controversial what role uric acid (UA) plays in heart failure. Conflicting current studies' findings suggest a reciprocal causality of the relationship between SUA elevation and CVD (17–20). Lowering uric acid alone may not improve the prognosis of patients with heart failure (21). In large cohort studies of the general population, associations between the whole range levels of SUA and prognosis supported a U-shaped association (22, 23). Epidemiological studies have suggested that hyperuricemia is a clinically significant risk factor or marker of lifestyle-related diseases that are the harbingers of HF such as hypertension, CKD, cerebrocardiovascular disease, metabolic syndrome (24–27). Hypoxemia due to HF may increase xanthine oxidase activity in cardiomyocytes and endothelial cells, resulting in increased SUA levels. Furthermore, Diuretic drugs are commonly used to relieve clinical HF symptoms, and they can induce hyperuricemia through increasing UA reabsorption and decreasing UA secretion. It is intuitive to suspect a relationship between UA and HF, and hyperuricemia is indeed related to worse prognosis in HF patients (28).

Patients with hypouricemia were characterized by older age, women, low BMI, non-smokers, statins, and diabetes. Patients with hyperuricemia were characterized by male sex, dyslipidemia, dilated cardiomyopathy, atrial fibrillation, history of HF admission, use of diuretics, low LVEF, and sizeable left ventricle (28). We performed genetic matching to equilibrate these covariates to output a more reliable analysis.

High SUA was found to be an independent prognosticator of adverse outcomes of HF outpatients in cohort studies (8, 9). PARAGON-HF showed that sacubitril–valsartan treated HFpEF patients had the lowest incidence rates of the primary composite endpoint, cardiovascular death, heart failure hospitalization, all-cause death, myocardial infarction, and stroke, with baseline SUA levels around 4–6 mg/dl (238–357 μmol/L) (29). Our results indicated that SUA has a U-shaped relationship with the HHF with a similar SUA concentration. The mechanisms of the relation between low UA levels and HHF remain to be elucidated.

### Lipoproteins and Blood Sugar

Freeman et al. showed that the deposition of cholesterol-rich microdomains in the macrophage's surface and the ECM resulting from acetylated (Ac)-LDL loading could be inhibited by the presence of ApoA-I (the major component of HDL particles) *in vitro* (30). Low-density lipoprotein cholesterol (LDL-c), high-density lipoprotein cholesterol (HDL-c), and triglycerides were regarded as predictors of outcomes in HF patients (31–33). Yet, a recent study showed that LDL-c was not associated with cardiovascular outcomes in overweight or obese subjects (34). We also did not detect a significant difference of LDL-c between hypertension and HHF.

Hyperglycemia is a conventional status in HF patients. Heart failure is associated with systemic insulin resistance due to insulin resistance, lack of movement, or exercise. Insulin resistance may lead to HDL dysfunction (35). HF patients with glucose metabolism alterations are more likely to be hospitalized than patients free from diabetes mellitus (36).

HDL is envisioned as a scavenger molecule that can reverse cholesterol transport and has anti-inflammatory, anti-oxidative, immunomodulatory, and endothelial protective properties. These properties are highly dependent on the proteome and lipidome of the particles (such as apolipoprotein A-I) and do not necessarily correlate with HDL-c levels. Low HDL or ApoA-I levels independently correlate with worsening prognosis in advanced HF (34, 37). Apolipoprotein M (apoM) is a multifunctional lipid mediator on HDL that was observed to be negatively correlated with BMI and the insulin resistance index, suggesting that apoM exerts protective roles against the development of insulin resistance (38). Studies *in vivo* with established heart failure have demonstrated that recombinant HDL_Milano_ induces regression of interstitial fibrosis and normalizes lung weight in mice with established heart failure (39). Taken together, HDL appears to play a substantial role in the entire progression of HHF. The addition of FBS and HDL-c to the model made our results of SUA-HHF correlation more reliable.

### Limitations

Several limitations of our study should be mentioned. First, the sample size after matching is relatively small, so the results should be carefully interpreted, for a small sample size has more chances to yield false-negative results. Secondly, we did not include arrhythmia and NYHA I patients in the matching, and atrial fibrillation is the most frequent cardiac complication in hypertensive patients. Lastly, we did not evaluate and stratify the patients based on the ejaculate fraction. These can lead to potential underlying bias and need caution generalizing the present findings.

## Conclusion

Our results supported a U-shaped causal association between serum uric acid levels and hypertensive heart failure, which means both low and high uric acid levels were predictive of hypertensive heart failure.

## Data Availability Statement

The original contributions presented in the study are included in the article/Supplementary Material, further inquiries can be directed to the corresponding author.

## Ethics Statement

The studies involving human participants were reviewed and approved by Ethics Committee of the Beijing Hospital (No. 2021BJYYEC-160-02). Written informed consent for participation was not required for this study in accordance with the national legislation and the institutional requirements.

## Author Contributions

HX was the major contributor to writing. YL and QW were involved in data collecting. DL made substantial contributions to research conception and designed the draft of the research process. HL gives technical support in the statistical methods. All authors read and approved the final manuscript.

## Funding

The Chinese Academy of Medical Sciences funded the present study, CAMS Innovation Fund for Medical Sciences (Grant No. 2018-I2M-1-002), National Key R&D Program of China (Grant No. 2020YFC2003000), and National Natural Science Foundation of China (Grant Nos. 31271097 and 51672030).

## Conflict of Interest

The authors declare that the research was conducted in the absence of any commercial or financial relationships that could be construed as a potential conflict of interest.

## Publisher's Note

All claims expressed in this article are solely those of the authors and do not necessarily represent those of their affiliated organizations, or those of the publisher, the editors and the reviewers. Any product that may be evaluated in this article, or claim that may be made by its manufacturer, is not guaranteed or endorsed by the publisher.
