# Supplementary figures and images for: U-Shaped Association Between Serum Uric Acid Level and Hypertensive Heart Failure: A Genetic Matching Case-Control Study
Source: Front Cardiovasc Med. 2021 Dec 8;8:708581. doi: 10.3389/fcvm.2021.708581 (PMC8692761; doi:10.3389/fcvm.2021.708581)

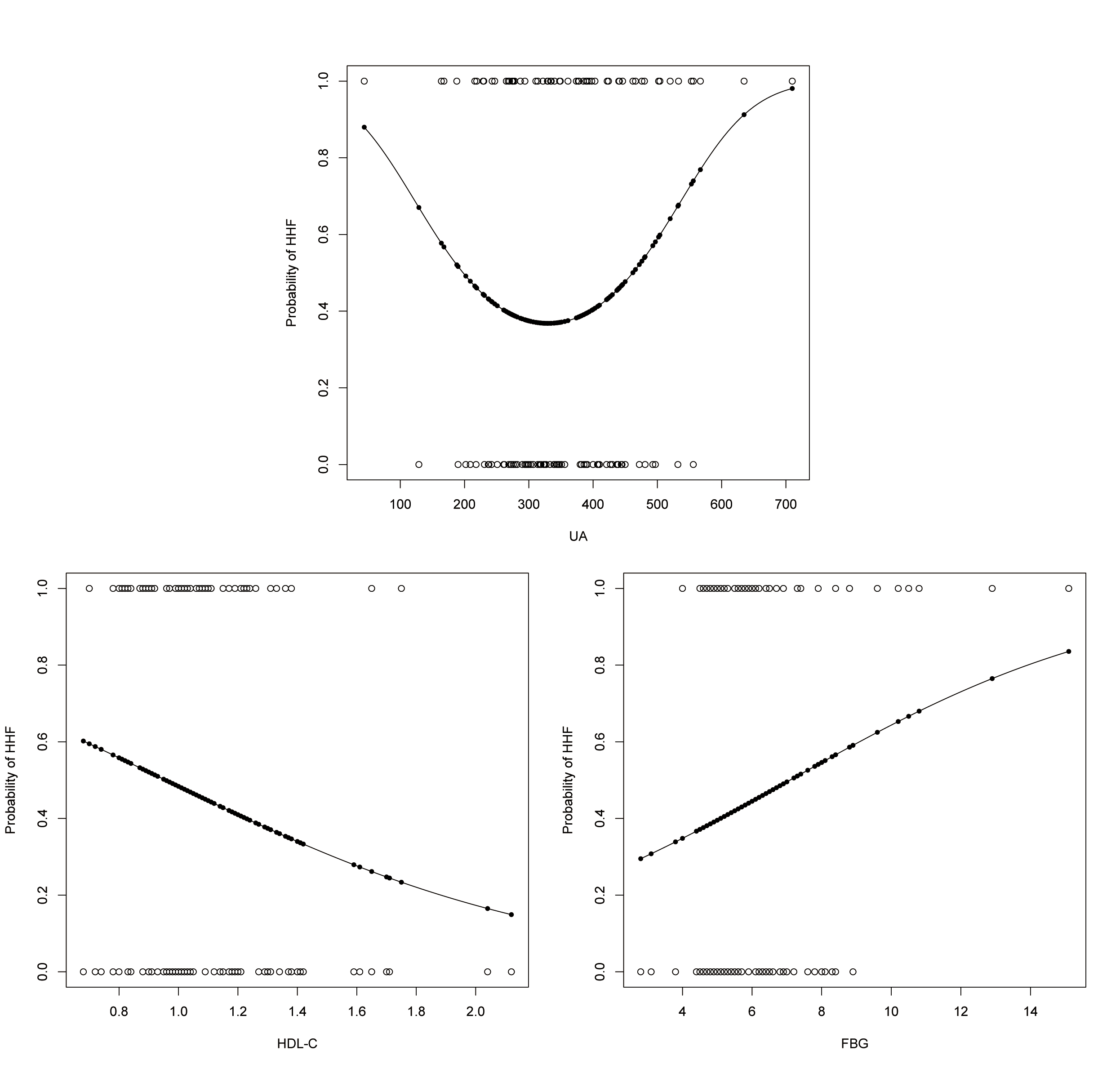

Supplement: Supplementary file 1 [file Image_1.TIF]
